# Meta-analysis of valved conduits in right ventricular outflow tract reconstruction: comparison of homograft, bovine jugular vein, and EPTFE valved conduits

**DOI:** 10.1097/JS9.0000000000004839

**Published:** 2026-01-19

**Authors:** Rui Wang, Huifeng Zhang, Ming Ye, Yaping Mi, Jiaxi Huang, Na Jiang, Li Guan, Zhizhou Shen, Yaping Shan

**Affiliations:** a Center for Clinical Practice Guideline Development and Evaluation, Children’s Hospital of Fudan University, Shanghai, China; b Department of Cardiovascular Center, Children’s Hospital of Fudan University, Shanghai, China

**Keywords:** bovine jugular vein conduits, homograft, polytetrafluoroethylene valved conduits, prognosis, right ventricular outflow tract reconstruction

## Abstract

**Background:**

Homograft, bovine jugular vein (BJV) and expanded polytetrafluoroethylene (ePTFE) valved conduits are most common valved conduits for reconstructing right ventricular outflow tracts (RVOT), while the performance of those three valved conduits was still unclear.

**Materials and methods:**

We searched Ovid MEDLINE, Ovid Embase, as well as Chinese databases of SinoMed, CNKI, and Wanfang from 1 January 2000 to 26 August 2024 to identify studies on RVOT reconstruction with homograft, BJV, and ePTFE valved conduits. We included studies reporting the outcomes of mortality, replacement rate, and the incidence of infective endocarditis. The “meta” and “metafor” packages in R version 4.2.1 were used for evidence synthesis. ROBINS-I tool was used for assessing the risk of bias. This study is registered on PROSPERO (CRD42024582184).

**Results:**

According to 28 retrospective cohort studies (*n* = 3966), there was no significant difference in mortality and replacement rate between homograft, BJV, and ePTFE. However, the IE incidence in BJV was significantly higher than that in homograft (RR = 3.63, 95% CI: 1.69–7.83, *P* < 0.01). Based on the pooled results of 113 case-series reports (*n* = 16 367), the total mortality of homograft, BJV, and ePTFE was 8% (95% CI: 6%–9%), 5% (95% CI: 4%–7%), and 3% (95% CI: 3%–4%), respectively. The replacement rate of homograft, BJV, and ePTFE was 9% (95% CI: 6%–13%), 9% (95% CI:6%–12%), and 4% (95% CI: 2%–9%), respectively. The incidence of IE for BJV was 6% (95% CI: 3%–10%), which was higher than 2% for homograft (95% CI: 1%–4%) and 1% for ePTFE (95% CI: 1%–2%).

**Conclusions:**

While BJV and ePTFE are viable alternatives with comparable mortality and replacement rate to homograft, the elevated IE risk in BJV necessitates cautious patient selection and monitoring.

## Introduction

The right ventricular outflow tract (RVOT), serving as the anatomical bridge between the right ventricle and pulmonary artery, plays a critical role in maintaining right ventricular hemodynamics^[^1^]^. Structural anomalies of the RVOT, frequently observed in congenital heart diseases (CHD), may lead to progressive right ventricular dysfunction^[^2^]^. Valved conduits have become indispensable in RVOT reconstruction for complex CHD repairs, including truncus arteriosus (TA), pulmonary atresia, and the ROSS procedure for aortic valve disease^[^3^]^. While Tetralogy of Fallot represents the most prevalent complex CHD, its surgical management has historically relied on non-valved techniques (e.g., trans-annular patch repair) due to limited availability of valved conduits and evolving therapeutic paradigms^[^4^]^. However, accumulating evidence from mid- to long-term follow-up studies reveals that such approaches predispose patients to significant pulmonary regurgitation and subsequent right ventricular remodeling^[^4^]^. This clinical dilemma underscores the persistent necessity of valved conduits for achieving durable RVOT reconstruction. Consequently, there remains an urgent demand for optimized valved conduit solutions in contemporary congenital cardiac surgery.

HIGHLIGHTS

- Bovine jugular vein (BJV) and expanded polytetrafluoroethylene (ePTFE) valved conduits are both good substitutes for the homograft.
- There is no difference in mortality, replacement rate among homograft, BJV and ePTFE for right ventrical outflow tract (RVOT) reconstruction.
- The risk for infective endocarditis is higher for BJV compared to homograft and ePTFE when it comes to RVOT reconstruction.
- This meta-analysis provided the pooled single proportions of mortality and incidence of complications after RVOT reconstruction with homograft, BJV, and ePTFE bases on 113 case-series reports involving 16 367 individuals.

The valved conduits can be divided into three main types: homograft (human donation), bioprosthetic valved conduit (synthesized from animal tissue), and polymer valved conduit (synthesized with high polymer material)^[^5^]^. Since valved conduits are not available in all types in cardiac medical centers, and most of current studies report the follow-up results of a single valved conduit type, this meta-analysis is crucial to compare the performance among different valved conduits. Although previous meta-analyses have been conducted, data on the prognostic outcomes of specific valved conduits remain limited^[^6^]^. To address this gap, our meta-analysis compares the outcomes of homograft, bovine jugular vein (BJV) conduits, the most commonly used bioprosthetic valved conduit in clinical practice, and expanded polytetrafluoroethylene (ePTFE) valved conduits, the most widely utilized polymer-based valved conduit. This study aims to evaluate the mortality, the replacement incidence, and the infective endocarditis (IE) incidence among patients receiving RVOT with BJV, homograft, and ePTFE. This study complies with the TITAN Guidelines 2025-governing declaration and use of AI^[^7^]^.

## Materials and methods

This systematic review adhered to the Preferred Reporting Items for Systematic Reviews and Meta-Analyses 2020 (PRISMA 2020) guidelines^[^8^]^ and was prospectively registered on the International Prospective Register of Systematic Reviews (PROSPERO ID: CRD42024582184). This systematic review is reported in line with AMSTAR (Assessing the methodological quality of systematic reviews) Guidelines^[^9^]^. The overall quality of the review was assessed using the AMSTAR-2 criteria^[^10^]^.

### Literature search

Appropriate keywords related to RVOT and valved conduits of interest were chosen through MeSH terms (in Medline), Emtree terms (in Embase), and their synonyms, along with consultation with subject matter experts and an overview of related articles aided in keyword selection. The systematic literature search was then conducted in Ovid MEDLINE, Ovid Embase, as well as Chinese databases of SinoMed, CNKI, and Wanfang from 1 January 2000 to 26 August 2024. Detailed search strategy can be found in the Supplemental Digital Content Material 1, available at: http://links.lww.com/JS9/G733.

### Study selection

Eligible studies should fulfill all of the following criteria: (1) focusing on patients undergoing RVOT reconstruction using valved conduits of BJV, homograft, or ePTFE and (2) reporting at least one of our primary outcomes (mortality and the incidence of conduit explantation or replacement). We excluded editorials, correspondence, reviews, case reports, and studies failing to report any specified outcomes for patients receiving RVOT reconstruction with BJV, homograft, or ePTFE valved conduits. Retrieved literature was first screened by titles and abstracts and then by full texts for eligibility by researchers (R.W. and H.Z.) independently. If necessary, the third researcher was consulted to make the final decision. Additionally, the reference lists of all included studies were reviewed to identify other eligible reports.

### Data extraction

We used a self-designed extraction sheet to collect the study design, first author, year of publication, country, the number of included participants for each valved conduits, and the diameter of valved conduits. We also extracted data on participant demographics, length of follow-up period, and outcomes related to mortality, incidence of replacement, or IE. To ensure accuracy, authors (R.W., H.Z., and Y.S.) independently extracted data. Extracted data were compared, with any discrepancies being resolved through discussion.

## Primary outcomes and secondary outcomes

Primary outcomes were mortality and incidence of replacement of valved conduits. Early mortality was defined as deaths within 30 days of RVOT reconstruction surgery or in-hospital deaths after the surgery. Late mortality referred to deaths after 30 days of the surgery or at discharge. Secondary outcomes referred to the IE incidence.

### Risk of bias assessment and certainty of evidence

Two authors (Y.S., H.Z.) independently assessed the quality of studies and risk of bias using the ROBINS-I tool for non-randomized controlled studies (Supplemental Digital Content Figure S1, available at: http://links.lww.com/JS9/G726). For evaluation of the certainty of evidence for each outcome, we used the Grading of Recommendations Assessment, Development, and Evaluation (GRADE; Supplemental Digital Content Material S2, available at: http://links.lww.com/JS9/G734; Supplemental Digital Content Material S3, available at: http://links.lww.com/JS9/G735; and Supplemental Digital Content Material S4, available at: http://links.lww.com/JS9/G736). Any discrepancies in judgments of risk of bias or justifications for judgments were resolved by discussion to reach consensus between the two review authors, with a third review author acting as an arbiter if necessary.

### Statistical analysis

Continuous variables were expressed as mean ± standard deviation or median with interquartile range. Relative risk (RR) or hazard ratio (HR) was used to present the comparison between valved conduits. For studies reporting a Kaplan-Meier survival curve for replacement or explantation of valved conduit with no reported HR, the HR was calculated according to the method reported by Jayne F Tierney^[^11^]^. A meta-analysis was conducted only when data were available from a minimum of two distinct studies.

To account for differences in study design and level of evidence, retrospective cohort studies and case series were aggregated separately for comparative analysis and pooled single-arm proportions. A random-effect model was employed, primarily because this model accounts for variations among the included studies and provides a more conservative estimation of the overall effect size. Statistical significance was set as *P* < 0.05. All statistical analyses were carried out using the “meta” and “metafor” packages in R version 4.2.1 (R Foundation for Statistical Computing).

Sensitivity analysis was conducted using a leave-one-out approach, sequentially excluding each study to evaluate the influence on the overall effect size. Consistency in the magnitude of effect estimates across these analyses was taken as evidence of robustness. Heterogeneity was assessed using forest plots and the *I*^2^ statistic and further explored via subgroup and risk factor analysis. Subgroup analysis was conducted when at least two studies were available, stratified by patient age, underlying diagnosis, and operative method. Risk factor analysis was restricted to variables with reported effect estimates (e.g., HR) in at least two studies. Multivariate effect sizes were prioritized for pooling, and univariate estimates were used only when multivariate data were unavailable. Publication bias was assessed using Egger’s test and funnel plots for outcomes with at least 10 studies (Supplemental Digital Content Figure S2, available at: http://links.lww.com/JS9/G727; Supplemental Digital Content Figure S3, available at: http://links.lww.com/JS9/G728; Supplemental Digital Content Figure S4, available at: http://links.lww.com/JS9/G729; Supplemental Digital Content Figure S5, available at: http://links.lww.com/JS9/G730).

## Results

We identified 6570 reports through database search. After removing duplication and screening for eligibility criteria, 141 reports remained for the analysis including 28 retrospective cohort studies and 113 case series reports. The PRISMA flowchart depicts an overview of the literature screening.

### Characteristics of included studies

These 141 reports included 20 333 patients receiving RVOT surgery. Among 28 retrospective cohort studies (*n* = 3966), 22 (78.6%) are for BJV vs homograft, 4 (14.3%) for ePTFE vs homograft, and 2 (7%) for ePTFE vs BJV. For 113 case series reports (*n* = 16,367), there are 62 (54.9%) for homograft, 32 (28.3%) for BJV and 19 (16.8%) for ePTFE. The baseline information of different valved conduits was summarized by Table 1. A comprehensive summary of study characteristics was presented in Supplemental Digital Content Table S1, available at: http://links.lww.com/JS9/G732.

**Table 1:** Baseline information of different valved conduits.

	Number of studies	Number of patients (female%)	Age, median (IQR), years	Follow-up time, median (IQR), years	Conduit diameter, mean ± SD, mm
BJV vs homograft	22	1325 (56.5%) vs 2128 (39.8%)	3 (0.2, 17) vs 3 (0.1, 25.7)	4.4 (1.7, 7.1) vs 7.5 (1.4, 13.6)	16 ± 3.2 vs 19 ± 6.8
ePTFE vs homograft	4	209 (31.3%) vs 125 (39.8%)	5.7 (1.1, 10.3) vs 6 (1.8,10.3)	2.9 (0.4, 5.4) vs 4.7 (0.6, 8.8)	17.9 ± 4.4 vs 18.7 ± 6.1
ePTFE vs BJV	2	46 (47.8%) vs 74 (41.9%)	4.1 (0.2, 7.9) vs 4.3 (0.6, 8.4)	3.1 (1.4, 4.8) vs 6.7 (2.2, 11.2)	/
Homograft	62	9867 (34.8%)	22.5(10.9, 34.1)	8 (4.2, 11.9)	22.7 ± 4.3
BJV	32	2832 (43.4%)	7.1(1, 13.3)	3.8 (2, 5.6)	17.6 ± 3.7
ePTFE	19	3668 (50.2%)	6.6(0, 13.4)	3.4 (1.5, 5.4)	17.8 ± 3.2

BJV, bovine jugular vein; ePTFE, expanded polytetrafluoroethylene; IQR, interquartile range; SD, standard deviation.

### Comparison between valved conduits

#### BJV vs homograft

**Figure 1.**
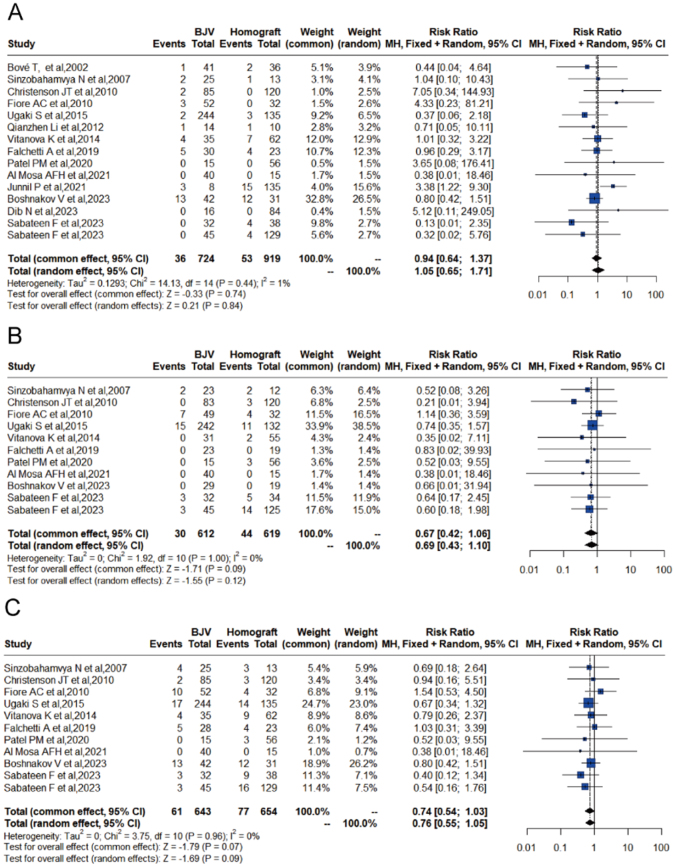
Forest plot of mortality of RVOT reconstruction with BJV vs homograft. Pooled rate ratio (RR) and 95% CI for early mortality (A), late mortality (B), and overall mortality (C) of RVOT reconstruction with BJV vs homograft. BJV, bovine jugular vein; RVOT, right ventricular outflow tract; ePTFE, expanded polytetrafluoroethylene; RR, risk ratio.

**Figure 2.**
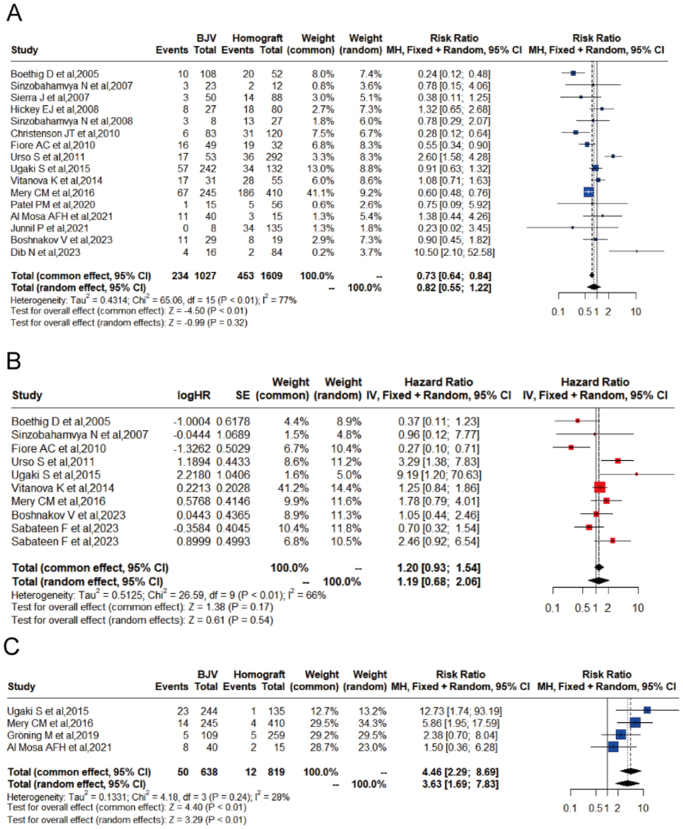
Forest plot of replacement and infective endocarditis of RVOT reconstruction with BJV vs homograft. Pooled RR (A), HR (B) for replacement rate, and pooled RR for infective endocarditis (C) of RVOT reconstruction with BJV vs homograft. BJV, bovine jugular vein; RVOT, right ventricular outflow tract; HR, hazard ratio; RR, risk ratio.

Based on pooled results from retrospective cohort studies comparing BJV and homograft, there was no significant difference in early mortality (RR = 1.05, 95% CI: 0.65–1.71, *P* = 0.84, *I*^2^ = 1%, Fig. 1A), late mortality (RR = 0.69, 95% CI: 0.43–1.10, *P* = 0.12, *I*^2^ = 0%, Fig. 1B), overall mortality (RR = 0.76, 95% CI: 0.55–1.05, *P* = 0.09, *I*^2^ = 0%, Fig. 1C), and replacement rate (RR = 0.82, 95% CI: 0.55–1.22, *P* = 0.32, *I*^2^ = 77%, Fig. 2A). Considering the potential influence of follow-up duration, a further analysis of HR was conducted from 10 studies, also showing no significant difference (HR = 1.19, 95% CI: 0.68–2.06, *P* = 0.54, *I*^2^ = 66%, Fig. 2B). However, regarding the IE incidence, pooled data from four studies demonstrated a significantly higher risk in the BJV group compared to the homograft group (RR = 3.63, 95% CI: 1.69–7.83, *P* < 0.01, *I*^2^ = 28%, Fig. 2C).

#### ePTFE vs homograft

**Figure 3.**
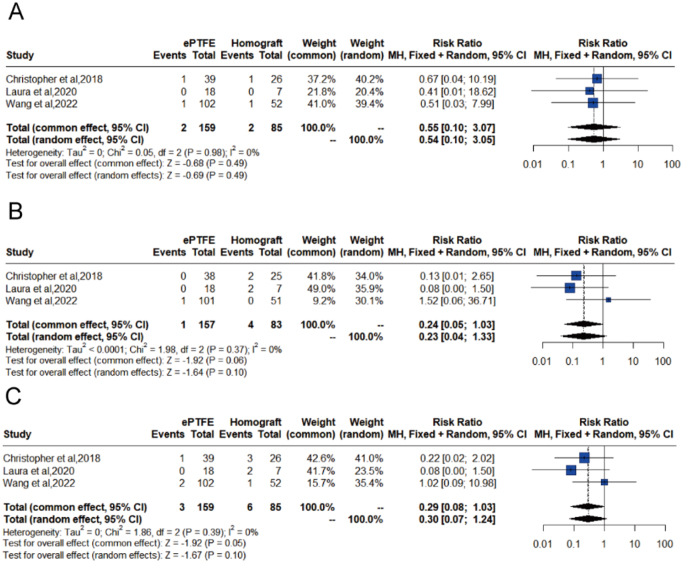
Forest plot of mortality of RVOT reconstruction with ePTFE vs homograft. Pooled RR and 95% CI for early mortality (A), late mortality (B), and total mortality (C) of RVOT reconstruction with ePTFE vs homograft. RVOT, right ventricular outflow tract; ePTFE, expanded polytetrafluoroethylene; RR, risk ratio.

**Figure 4.**
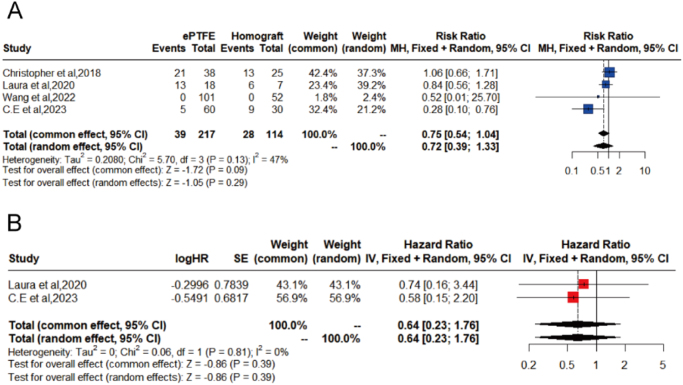
Forest plot of replacement of RVOT reconstruction with ePTFE vs homograft. Pooled RR (A) and HR (B) for the replacement rate of reconstruction RVOT with ePTFE vs homograft. RVOT, right ventricular outflow tract; ePTFE, expanded polytetrafluoroethylene; RR, risk ratio; HR, hazard ratio.

Three retrospective cohort studies reported mortality outcomes between the ePTFE and homograft groups. The pooled results showed no significant differences in early mortality (RR = 0.54; 95% CI: 0.10–3.05; *P* = 0.49; *I*^2^ = 0%), late mortality (RR = 0.23; 95% CI: 0.04–1.33; *P* = 0.06; *I*^2^ = 0%), and overall mortality (RR = 0.30; 95% CI: 0.07–1.24; *P* = 0.10; *I*^2^ = 0%) between the two groups (Fig. 3A-C). Pooled data from four retrospective cohort studies comparing replacement rates demonstrated no statistically significant difference (RR = 0.75; 95% CI: 0.54–1.04; *P* = 0.09; *I*^2^ = 47%; Fig. 4A). The pooled HR from two retrospective cohort studies also indicated no significant difference (HR = 0.64; 95% CI: 0.23–1.76; *P* = 0.39; *I*^2^ = 0%; Fig. 4B). IE incidence was not reported in any of the included studies comparing ePTFE with homograft.

#### Expanded polytetrafluoroethylene vs bovine jugular vein

**Figure 5.**
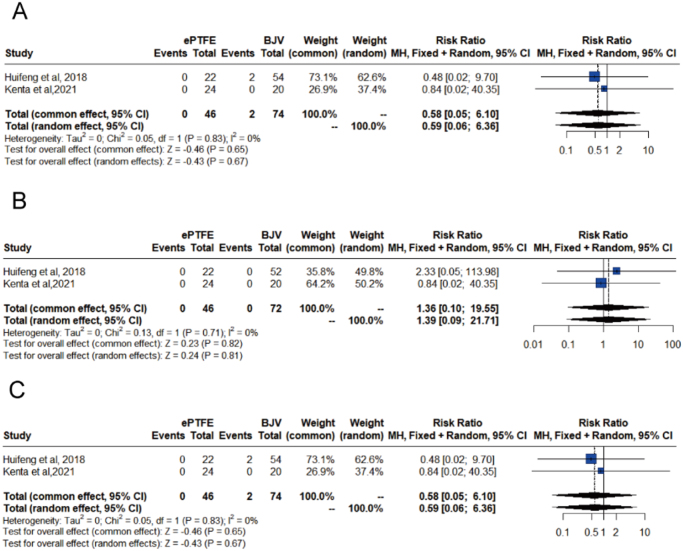
Forest plot of mortality of RVOT reconstruction with ePTFE vs BJV. Pooled RR and 95% CI for early mortality (A), late mortality (B), and total mortality (C) of reconstruction RVOT with ePTFE vs BJV. RVOT, right ventricular outflow tract; ePTFE, expanded polytetrafluoroethylene; BJV, bovine jugular vein; RR, risk ratio.

**Figure 6.**
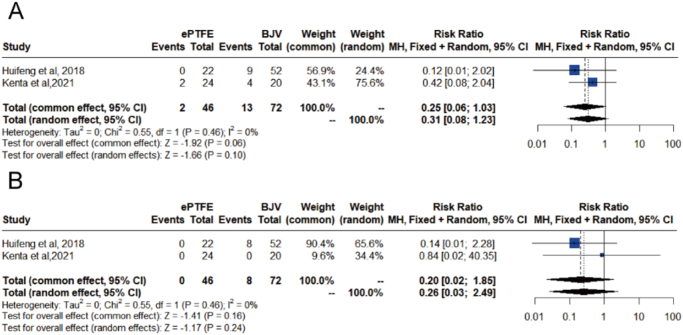
Forest plot of replacement and infective endocarditis of RVOT reconstruction with ePTFE vs BJV. Pooled RR (A) for replacement rate, and infective endocarditis (B) of reconstruction RVOT with ePTFE vs BJV. RVOT, right ventricular outflow tract; ePTFE, expanded polytetrafluoroethylene; BJV, bovine jugular vein; RR, risk ratio.

A meta-analysis of two retrospective cohort studies demonstrated no significant differences between the ePTFE and BJV groups in terms of early mortality (RR = 0.59; 95% CI: 0.06–6.36; *P* = 0.67; *I*^2^ = 0%, Fig. 5A), late mortality (RR = 1.36; 95% CI: 0.10–19.55; *P* = 0.82; *I*^2^ = 0%, Fig. 5B), and overall mortality (RR = 0.59; 95% CI: 0.06–6.36; *P* = 0.67; *I*^2^ = 0%, Fig. 5C). No significant differences were observed between the two groups regarding the replacement rate (RR = 0.31; 95% CI: 0.08–1.23; *P* = 0.10; *I*^2^ = 0%, Fig. 6A) and the IE incidence (RR = 0.26; 95% CI: 0.03–2.49; *P* = 0.24; *I*^2^ = 0%, Fig. 6B).

## Pooled outcomes for individual valved conduit

### Homograft

**Figure 7.**
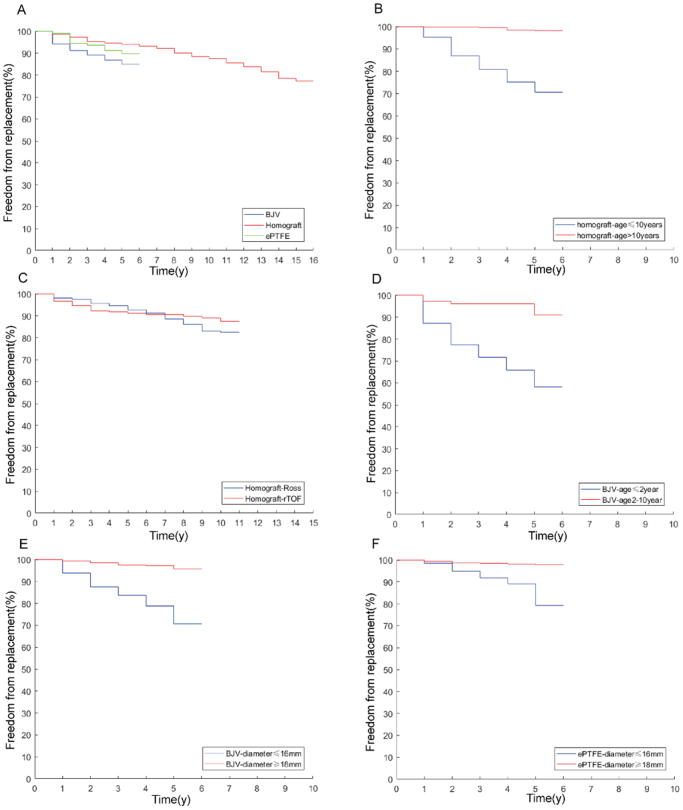
Kaplan-Meier curves of freedom from replacement. Freedom from replacement after RVOT reconstruction with homograft, BJV, and ePTFE. (B) Freedom from replacement in subgroups of “age ≤10 years” and “age >10 years” in reconstructing RVOT with homograft. (C) Freedom from replacement in subgroups of “ROSS” and “r-TOF” in reconstructing RVOT with homograft. (D) Freedom from replacement in subgroups of “age ≤2 years” and “age 2–10 years” in reconstructing RVOT with BJV. (E) Freedom from replacement in subgroups of “conduit diameter ≤16 mm” and “conduit diameter ≥18 mm” in RVOT reconstruction with BJV. (F) Freedom from replacement in subgroups of “conduit diameter ≤16 mm” and “conduit diameter ≥18 mm” in reconstructing RVOT with ePTFE. RVOT, right ventricular outflow tract; ePTFE, expanded polytetrafluoroethylene; BJV, bovine jugular vein; r-TOF, repaired Tetralogy of Fallot.

The pooled estimated proportions of early mortality, late mortality, and overall mortality for patients using homograft were 4% (95% CI: 2%–5%, *I*^2^ = 85%), 4% (95% CI: 3%–5%, *I*^2^ = 69%), and 8% (95% CI: 6%–9%, *I*^2^ = 82%), respectively (Table 2). Additionally, the pooled estimated proportion of replacement rate was 9% (95% CI: 6%–13%, *I*^2^ = 95%). As shown in Figure 7A, the freedom from replacement at 5, 10, and 15 years for all patients was 94.0% (range: 90.4%–98.8%), 87.5% (range: 82.0%–95.9%), and 77.4% (range: 61.0%–92.5%), respectively. The primary causes of homograft replacement were stenosis (64.3%), combined stenosis and regurgitation (15.0%), and regurgitation (12%) as detailed in Table 3. The pooled estimated proportion of IE incidence was 2% (95% CI: 1%–4%, *I*^2^ = 68%; Table 2).

**Table 2:** The pooled rate for a single valved conduit.

		Homograft	BJV	ePTFE
Early mortality	Number of studies	47	31	19
	Events/total	274/7805	95/2638	58/3660
	Rate (95% CI)	4% (2%–5%)	3% (2%–4%)	1% (1%–2%)
	I^2^	85%	71%	12%
Late mortality	Number of studies	45	31	18
	Events/total	325/7011	75/2537	66/3583
	Rate (95% CI)	4% (3%–5%)	2% (1%–3%)	2% (1%–2%)
	I^2^	69%	43%	24%
Total mortality	Number of studies	58	32	18
	Events/total	714/9131	185/2815	124/3641
	Rate (95% CI)	8% (6%–9%)	5% (4%–7%)	3% (2%–4%)
	I^2^	82%	71%	35%
Replacement	Number of studies	52	32	17
	Events/total	1024/8355	368/2766	275/3557
	Rate (95% CI)	9% (6%–13%)	9% (6%–12%)	4% (2%–9%)
	I^2^	95%	81%	92%
Infective endocarditis	Number of studies	9	8	6
	Events/total	41/1755	66/1078	10/886
	Rate (95% CI)	2% (1%–4%)	6% (3%–10%)	1% (0%–2%)
	I^2^	68%	83%	30%

BJV, bovine jugular vein; ePTFE, expanded polytetrafluoroethylene.

**Table 3:** Reasons of valved conduit replacement.

	Homograft (21)		BJV (19)		ePTFE (7)	
Reasons for replacement	Events/total	Ratio (%)	Events/total	Ratio (%)	Events/total	Ratio (%)
Stenosis of valved conduit	365/568	64.3	120/277	43.3	79/234	33.8
Regurgitation of valved conduit	68/568	12.0	14/277	5.1	12/234	5.1
Stenosis and regurgitation of valved conduit	85/568	15.0	44/277	15.9	NA	NA
Infective endocarditis	26/568	4.6	45/277	16.2	17/234	7.3
Somatic growth	2/568	0.4	22/277	7.9	77/234	32.9
Compression of valved conduit	NA	NA	1/277	0.4	NA	NA
Thrombus of valved conduit	NA	NA	5/277	1.8	NA	NA
Dilation of valved conduit	NA	NA	3/277	1.1	NA	NA
Aneurysm formation	10/568	1.8	8/277	2.9	NA	NA
Coronary compression	NA	NA	NA	NA	1/234	0.4
Other reasons	12/568	2.1	14/277	5.1	48/234	20.5

BJV, bovine jugular vein; ePTFE, expanded polytetrafluoroethylene.

Events/total = replacement due to one specific reason/total replacement.

Subgroup analysis revealed that both early mortality and the replacement rate were higher in younger patients and were the highest among those with TA (Table 4). The survival curves depicting freedom from replacement stratified by age subgroup and diagnostic subgroup are shown in Figure 7B and Figure 7C, indicating that younger age was associated with a higher replacement rate.

**Table 4:** Subgroup analysis for homograft.

		Early mortality	Late mortality	Total mortality	Replacement
Subgroup-age: <18 years	Number of studies	11	11	13	11
	Events/total	46/1277	44/1225	106/1445	143/1320
	Rate (95% CI)	4% (1%–7%)	3% (2%–4%)	8% (5%–12%)	8% (3%–16%)
	I^2^	84%	36%	76%	93%
Subgroup-age: <10 years	Number of studies	2	2	3	3
	Events/total	9/161	7/152	27/203	55/195
	Rate (95% CI)	6% (3%–10%)	4% (2%–8%)	15% (6%–28%)	15% (0%–44%)
	I^2^	0%	33%	68%	85%
Subgroup-age: < 2 years	Number of studies	2	2	2	4
	Events/total	17/120	10/103	27/120	74/277
	Rate (95%CI)	14% (9%–21%)	9% (5%–16%)	22% (15%–30%)	27% (18%–37%)
	I^2^	0%	25%	0%	59%
Subgroup-age: <1 years	Number of studies	4	4	4	/
	Events/total	31/235	18/204	49/235	/
	Rate (95% CI)	16% (8%–27%)	5% (0%–14%)	21% (16%–26%)	/
	I^2^	69%	74%	0%	/
Subgroup-diagnosis: ROSS	Number of studies	14	13	17	15
	Events/total	62/3748	119/3239	199/3795	153/3421
	Rate (95% CI)	1% (1%–2%)	4% (3%–4%)	5% (3%–6%)	4% (2%–7%)
	I^2^	77%	31%	66	89%
Subgroup-diagnosis: r-TOF	Number of studies	2	2	4	4
	Events/total	2/291	12/289	20/470	41/468
	Rate (95% CI)	0% (0%–3%)	4% (0%–12%)	4%(1%–8%)	9% (6%–11%)
	I^2^	77%	88%	72%	0%
Subgroup-diagnosis: Truncus	Number of studies	3	3	3	3
	Events/total	25/220	22/195	47/220	50/208
	Rate (95% CI)	14% (6%–24%)	11% (7%–16%)	21% (16%–27%)	24% (14%–35%)
	I^2^	61%	0%	15%	53%

r-TOF, repaired Tetralogy of Fallot.

### Bovine jugular vein

The pooled estimated proportions of early mortality, late mortality, and overall mortality was 3% (95% CI: 2%–4%, *I*^2^ = 71%), 2% (95% CI: 2%–3%, *I*^2^ = 43%), and 5% (95% CI: 4%–7%, *I*^2^ = 71%), respectively (Table 2). The pooled estimated proportions of replacement rate were 9% (95% CI: 6%–12%, *I*^2^ = 91%; Table 2). The 5-year freedom from replacement was 85.0% (range: 71.0%–97.0%, Figure 7A). The primary causes of BJV replacement were stenosis (43.3%), infection (16.2%), and combined stenosis and regurgitation (15.9%) as detailed in Table 3. The pooled estimated proportion of IE incidence after BJV implantation was 6% (95% CI: 3%–10 %, *I*^2^ = 83%; Table 2), the highest among the three valved conduits.

Subgroup analysis revealed that in patients younger than 18 years, the pooled estimated proportions of early mortality, late mortality, overall mortality, and replacement rate were 5% (95% CI: 2%–10%, *I*^2^ = 80%), 3% (95% CI: 2%–4%, *I*^2^ = 48%), 8% (95% CI: 4%–13%, *I*^2^ = 75%), and 6% (95% CI: 3%–10%, *I*^2^ = 72%), respectively. The survival curves for freedom from replacement stratified by age and conduit diameters are presented in Figure 7D and Figure 7E, indicating that younger age and smaller diameter are associated with a higher replacement rate.

### Expanded polytetrafluoroethylene

The pooled estimated proportions of early mortality, late mortality and overall mortality was 1% (95% CI: 1%–2%, *I*^2^ = 12%), 2% (95% CI: 1%–2%, *I*^2^ = 24%), and 3% (95% CI: 3%–4%, *I*^2^ = 35%), respectively (Table 2). The pooled estimated replacement rate was 4% (95% CI: 2%–9%, *I*^2^ = 92%; Table 2). The 5-year freedom from replacement rate was 89.8% (range: 81.7%–100%, Fig. 7A). The primary causes of ePTFE replacement were mismatch by somatic growth (32.9%), stenosis (31.2%), and operation (19.2%; Table 3). The pooled estimated proportions of IE incidence of was 1% (95% CI: 0%–2%, *I*^2^ = 30%; Table 2).

Subgroup analysis revealed that in patients younger than 18 years, pooled estimated proportions of early mortality, late mortality, total mortality, and replacement rate was 2% (95% CI: 1%–3%, *I*^2^ = 4%), 3% (95% CI: 1%–5%, *I*^2^ = 4%), 5% (95% CI: 3%–7%, *I*^2^ = 47%), and 3% (95% CI: 0%–20%, *I*^2^ = 87%), respectively. The survival curves for freedom from replacement stratified by conduit diameters are presented in Figure 7F, indicating that younger age was associated with a higher replacement rate.

### Sensitivity analysis

Leave-one-out sensitivity analysis confirmed the robustness of most pooled estimates (Supplemental Digital Content Material S5, available at: http://links.lww.com/JS9/G737). However, for the comparison of ePTFE versus homograft, the results for late and overall mortality were influenced by the inclusion of Wang *et al* (2022). While no significant differences were observed in the primary analysis, the exclusion of this study shifted the results to statistical significance for both late mortality and overall mortality. This substantial influence is attributable to the rare events and the limited number of included studies. Wang *et al* was the only study reporting a neutral or opposing effect direction (RR > 1.0), and given its relatively high weight in the random-effects model, its inclusion diluted the strong protective effects observed in the other two studies.

### Heterogeneity analysis

The comparative analysis based on retrospective cohort studies generally demonstrated low-to-moderate heterogeneity, indicating relatively consistent results across studies. In contrast, substantial heterogeneity was primarily observed in single-arm analyses where data from case series were pooled to estimate overall proportions. The heterogeneity may arise from the following aspects. First, surgical techniques varied across medical centers and evolved over different time periods. Second, baseline patient characteristics, including age, weight, and underlying medical conditions, varied among studies. Third, conduit-related factors such as the source of homograft, the manufacturing processes of BJV conduits, and the suturing techniques used for ePTFE conduits were not standardized. Fourth, differences in conduit-patient size matching (e.g., *Z*-scores) may have influenced conduit performance and durability. In addition, variations in postoperative management strategies, including anticoagulation and infection prophylaxis, as well as differences in follow-up duration, likely contributed further to outcome variability. Furthermore, the intrinsic limitations of case series – such as the absence of control groups, inconsistent inclusion criteria, and unstandardized outcome reporting – make them prone to heterogeneity. Moreover, evidence from pooled estimates of risk factors for conduit replacement also suggests that certain clinical and procedural factors may also influence between-study variability (Supplemental Digital Content Figure S6, available at: http://links.lww.com/JS9/G731). Younger age was associated with an increased risk of valved conduit replacement (HR = 2.44, 95% CI 1.72–3.47; *P* < 0.00001; *I*^2^ = 43%). A larger conduit diameter was associated with a reduced risk (HR = 0.69, 95% CI 0.55–0.88; *P* = 0.003; *I*^2^ = 92%). The use of an aortic homograft was also significantly associated with homograft failure (HR = 1.75, 95% CI 1.26–2.43; *P* = 0.0009; *I*^2^ = 12%).

## Discussion

The key finding of the meta-analysis showed that overall mortality did not differ across the three valved conduits, and replacement rates were comparable in the current evidence base. However, the incidence of IE was significantly higher in BJV.

The comparable mortality across conduit types suggests that survival after RVOT reconstruction is not influenced by the type of valved conduit. The subgroup analysis in our research revealed that patients aged less than 2 years or those diagnosed with TA had a higher mortality rate, which indicated that the survival after RVOT reconstruction may be influenced by age and diagnosis.

The dysfunction or failure of valved conduits was the most important postoperative complication, which can lead to the reintervention^[^12–14^]^. Reintervention includes catheter-based intervention and replacement of failure valved conduit. As the definition of conduit dysfunction, conduit failure and reintervention or catheter-based intervention varies, we did not set those as the outcome. According to our meta-analysis, there was no difference in the incidence of replacement between homograft, BJV, and ePTFE. However, the follow-up time of homograft was longer than that of BJV and ePTFE; therefore, whether this conclusion holds in long-term follow-up requires further verification. Our meta-analysis still showed that patients with a younger age and a smaller conduit diameter had a higher risk of valved conduit replacement. In addition, the *Z*-score, which indicates the degree of matching between the conduit and the patient, is also a significant determinant of conduit replacement. Previous studies reported that both overly high (*Z* ≥ 2.7^[^15^]^ or *Z* > 3^[^16^]^) and low *Z*-value (*Z* < 1^[^16,17^]^) pose risks, but a conclusive recommended range remains unclear. Another finding of our research was that the incidence of replacement after reconstructing RVOT with aortic homograft was about 1.75 times higher than that of pulmonary homograft, which is consistent with other studies^[^18–20^]^, indicating that aortic homograft was an independent risk factor for homograft failure. This may be because the aortic valve’s structure differs from that of the pulmonary valve^[^21^]^, so when transplanted from the high-pressure systemic circulation to the low-pressure pulmonary circulation, this structural and functional mismatch leads to hemodynamic abnormalities^[^22^]^. Furthermore, the thicker, more dense structure of the aortic wall and leaflets impedes host cell infiltration and new capillary formation, which hampers the clearance of necrotic tissue and increases susceptibility to immune responses and calcification^[^23^]^. The conduit stenosis was the predominant reason for replacement with homograft, BJV, and ePTFE. Pathological findings indicated that stenosis were caused by fibrosis and calcification of valved conduit^[^23–28^]^. Except for foreign body reaction or immune response, the fibrosis of BJV can be caused by glutaraldehyde remnant^[^26^]^. The mechanism of ePTFE calcification was proteinaceous infiltration^[^29^]^, different from homograft calcification, which was induced by high elastin tissue content^[^23^]^. Additionally, the latest research has found that environmental factors such as PM2.5 can also affect the function of the valved conduit^[^30^]^. There is no growth potential for ePTFE, homograft, and BJV^[^31^]^, and relative stenosis caused by patients’ growth was another critical reason for conduit reintervention. Our meta-analysis demonstrated that this constitutes the principal indication for ePTFE replacement; therefore, oversized valved conduits are often used in children^[^15^]^. Although homograft, BJV, and ePTFE have relatively good hemocompatibility and do not require lifelong anticoagulation or antiplatelet therapy, thrombosis remains a significant cause of stenosis, particularly in conduits with smaller internal diameters^[^32^]^. Consequently, many medical centers adopt a short-term anticoagulation and antiplatelet strategy^[^33–35^]^. However, the specific anticoagulation protocols vary between centers, and there is limited reporting on them in previous studies^[^36^]^. Therefore, randomized controlled trials to evaluate the anticoagulation regimen for each type of conduit are of great significance. With the extension of implantation time, the probability of degeneration and mismatch of conduit increases, and the freedom from replacement goes down every year both in homograft, BJV, and ePTFE. The development of catheter-based intervention such as percutaneous pulmonary valve implantation can avoid or delay the replacement operation^[^37^]^. Recently, there is ongoing research on valved conduits with good biocompatibility and growth potential^[^38,39^]^, which may decrease the incidence of reintervention in the future.

There is a risk of IE for any valved conduit implantation^[^38^]^. The IE incidence for BJV is the highest as shown in our meta-analysis, which may be attributable to the following reasons. First, BJV tissue can promote bacterial adhesion^[^39^]^. Second, the preparation of BJV conduits destroys the native tissue structure and eliminates the endothelial barrier, which facilitates flow turbulence and thrombus formation, thus increasing the risk of infection^[^40^]^. Third, daily predisposing factors such as dental and skin problems further increase the risk^[^26^]^. Therefore, antibiotic prophylaxis is essential not only in the pre- and post-operative periods but also after skin injuries and dental procedures. The incidence of IE increased with the duration of BJV implantation^[^40,41^]^. It should also be noted that the incidence of IE increases with the duration of BJV implantation, so patients who accept BJV valved conduits require lifelong multimodal imaging follow-up. Further research is necessary to investigate the optimal types and duration of prophylactic antibiotic regimens.

The homograft has the most extended history of clinical use and is unquestionably the first choice for physicians in reconstructing the RVOT. However, due to factors such as culture and religious beliefs, homograft is unavailable in some regions, or suitable sizes may be lacking. Our meta-analysis showed no significant differences in mortality and replacement rate among BJV, ePTFE, and homograft after implantation. Therefore, BJV and ePTFE can be considered as alternatives in cases of homograft scarcity. However, both BJV and ePTFE have their shortcomings. For instance, BJV carries a high risk of IE, while ePTFE is prone to quality issues due to suturing techniques. Therefore, it is recommended that clinicians select the appropriate conduit based on its availability and compatibility with the patient.

### Study limitations

Although this meta-analysis provides meaningful insights into the outcomes of RVOT reconstruction, several limitations warrant attention. First, all included studies were observational and mainly retrospective, which inevitably introduces selection bias and confounding factors inherent to non-randomized designs. Second, the limited number of studies for specific comparisons (e.g., ePTFE vs. homograft) and rare events could bring unstable estimates. Third, potential heterogeneity may arise from variations in clinical factors, such as follow-up duration, conduit origin, and fabrication methods. These inconsistencies may affect the precision of the pooled estimates and limit the generalizability of the findings. Fourth, our search focused on valved conduits of homograft, BJV, and ePTFE, and did not include other types (e.g., decellularized bioprosthetic valved conduits and porcine valved conduits). Additionally, although we reported major complications related to valved conduit implantation, data on less frequently reported adverse events were insufficient, limiting our ability to conduct a more comprehensive safety assessment.

Despite these limitations, this study represents the most current synthesis of evidence, offering valuable guidance for clinical decision-making and highlighting areas where further high-quality, prospective investigations are necessary.

## Conclusions

The meta-analysis of 141 studies revealed that RVOT reconstruction with homograft, BJV, and ePTFE valved conduits had comparable mortality and replacement rates, indicating that BJV and ePTFE valved conduits can be used as viable alternatives in case of homograft scarcity. However, the elevated IE risk associated with BJV warrants careful patient selection and vigilant long-term follow-up.

## Data Availability

This is a meta-analysis of previously published studies. All data analyzed are publicly available in the original publications.

## Research registration unique identifying number (UIN)

PROSPERO (CRD42024582184).

## Assistance with the study

During the revision process, the authors used ChatGPT 5.0 for grammar checks and language refinement. After using this tool, the authors reviewed and edited the content as needed and take full responsibility for the content of the publication.
